# A Primary Giant Hydatid Cyst of the Ovary

**DOI:** 10.5812/iranjradiol.7955

**Published:** 2012-09-17

**Authors:** Abdurrahman Kaya, Sibel Yildiz, Reşat Özaras, Ali Mert

**Affiliations:** 1 Department of Infectious Diseases, Medical School of Cerrahpasa, Istanbul University, Istanbul, Turkey

**Keywords:** Cysts, Ovary

**Figure 1:**
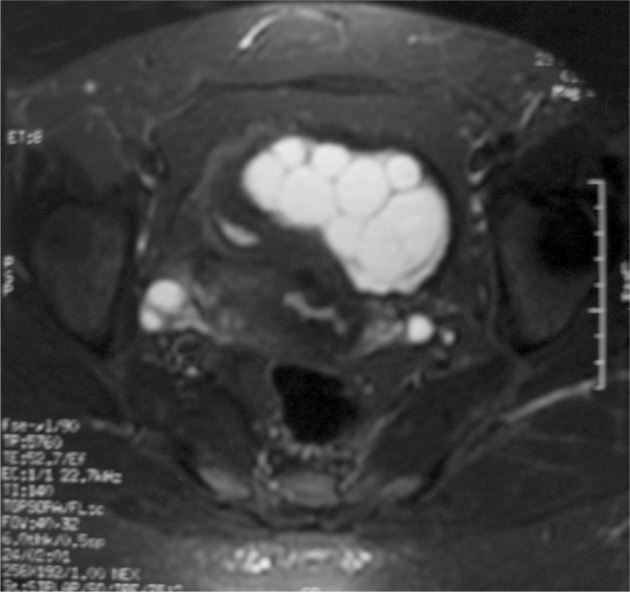
Pelvic MRI revealed a septated, lobulated and contrast enhancing cystic mass that is hyperintense on T2 and hypointense on T1 FLAIR sequences.

Dear Editor,

A 38-year-old female patient was admitted with right lower abdominal pain of 4 months duration. On her clinical examination, right lower abdominal tenderness was noted. Complete blood count including the numbers of eosinophils (80/mm^3^) was normal. Biochemistry was normal. An abdominal MRI showed an 11 × 9 × 5.5 cm sized cystic lesion in the right ovary surrounded by loculated fluid collection with septations (Figure 1). Echinococcal serology was positive (hydatid cyst antibodies by ELISA and indirect hemagglutination test dilutions up to 1/1280 were positive). Pathological examination of the material had a cuticle and germinative membrane of the hydatid cyst and around it chronic nonspecific inflammation leading to the formation of lymphoid follicles was seen. No other organ involvement was detected. Albendazole (10mg/kg/day) was prescribed. The cyst was removed surgically on the 30th day of albendazole treatment. Pathological examination of the cyst was compatible with hydatid cyst. The patient had regular follow-ups, with no recurrence of the cyst. Echinococcal disease is endemic in many countries (1). It involves multiple organs particularly the liver (50-70%) and the lung (20-30%), while primary involvement of the ovary is very rare (2). The liver and spleen cysts can rupture into the peritoneal cavity which is the way of secondary involvement (3). In our case, there were no cysts in other organs. Although ovarian cysts are usually single, this case showed multilocular cysts the treatment is surgical removal and albendazole is recommended before surgery.
